# Proposal of measurement of vertical larynx position at rest

**DOI:** 10.1016/S1808-8694(15)31328-8

**Published:** 2015-10-20

**Authors:** Osiris de Oliveira Camponês do Brasil, Rosiane Yamasaki, Sylvia Helena de Souza Leão

**Affiliations:** ^1^ Doutor em Medicina pela Universidade Federal de São Paulo - Escola Paulista de Medicina; 2 Instituiçã o: Serviço de Otorrinolaringologia/Cabeça e Pescoço do Hospital do Servidor Público do Estado de São Paulo

**Keywords:** larynx, position, measurement, adult

## Abstract

**Aim**: The purpose of this research is to propose a procedure to measure the vertical larynx position in the neck at rest in young adults without vocal complaint. **Study Design**: Transversal cohort study. **Material and method**: There were 68 subjects, aged between 18 to 44 years, 33 female and 35 male. The anatomical landmarks used for this research study were the right and left jaw angle (RJA and LJA), the centre of the cricoid arch cartilage (CC) and the centre of the sternal furculum (SF). In order to obtain the measures, the subjects were asked to be sitting still with their heads stretched up to the highest possible position. The devices used were a drawing compass and a 20-centimeter ruler. **Results**: The measurement procedure proved to be easy and it did not show any discomfort to the participants. There was no statistically significant difference between genders related to the vertical larynx position in the neck; however the women presented higher larynx position than men. The vertical larynx position was easily obtained and it seems to be a very interesting parameter to intra-subject clinical follow-up.

## INTRODUCTION

The larynx is suspended in the neck and it is located between the hyoid bone and the trachea. At birth, it is placed at a high neck position, inferiorly limited by the cricoid cartilage around cervical vertebrae C3 and C4, and it slowly and continuously goes down until the senescence^1^. In adults, cricoid cartilage is at the level of the 6^th^ cervical vertebra (C6) and the arch is palpable in vivo^2^. The larynx is shorter in women and children and it is placed more superiorly. This difference between genders is normally developed in puberty in men, when cartilages increase in size.^3^

Given that many muscle groups are attached to laryngeal cartilages, many forces may influence its position depending on the magnitude of the contraction. Laryngeal extrinsic muscles should be capable of elevating and lowering it at the neck, and vocal frequency variation is a direct acoustic consequence of this movement. Elevated laryngeal position shortens the vocal tract, which increases all frequencies of formants^4^. It is assumed that the low position of the larynx produces the opposite effect. Thus, larynx height has an important acoustic effect over the speakers' voice.

Another important aspect is related to the source itself. Physiologically, elevated larynx is strongly associated with the swallowing process, working as a sphincter valve, a mechanism of airway protection. Laryngeal lowering is associated with inspiration and glottic opening, probably including an abductor component in the laryngeal lowering movement. Thus, phonation goes from a compressed to a more fluid pattern of emission^5^.

The literature and clinical practice have shown that elevated laryngeal position is frequently associated with voices that have a strong component of tension, especially in functional manifestations1., 6.. Conversely, the low laryngeal position is associated with a softer and more comfortable production pattern. Thus, vertical laryngeal position may be considered a very interesting parameter for the clinical and therapeutic follow up of dysphonic patients. However, the techniques described for measuring the laryngeal vertical position are still complex and very uncommon in our clinical practice.

The present study intended to suggest a practical and quick way of measuring the vertical position of the larynx in the neck at rest in young adults without vocal complaint.

## MATERIAL AND METHOD

The study was approved by the Ethics and Research Committee, Escola Paulista de Medicina - Federal University of Sao Paulo, under protocol nº 1555/03. The study was carried out by the sector of Larynx and Voice, EPM - UNIFESP, in 2004.

**Figure 1:**
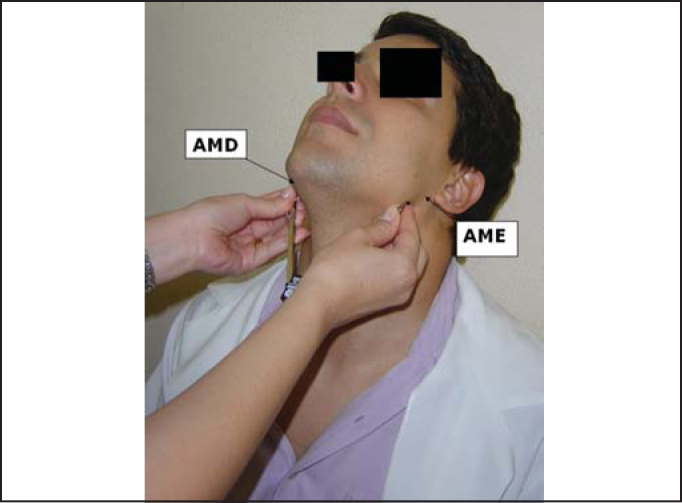
Marking of measurement of right mandible angle (AMD) to left angle (AME).

**Figure 2:**
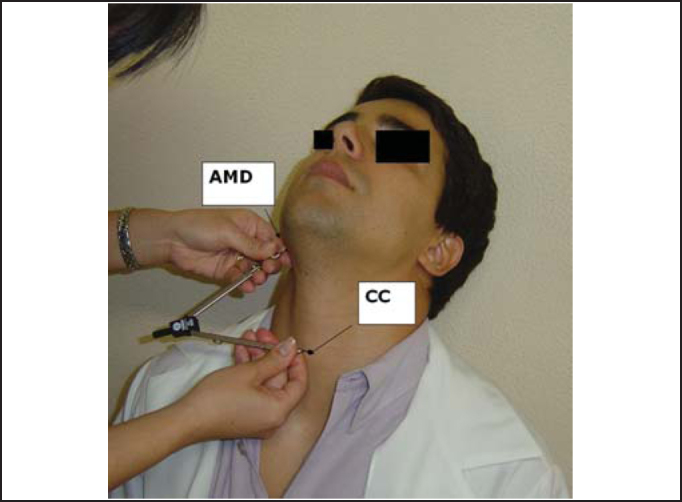
Marking of measurement of right mandible angle (AMD) to center of cricoid cartilage arch (CC).

**Figure 3:**
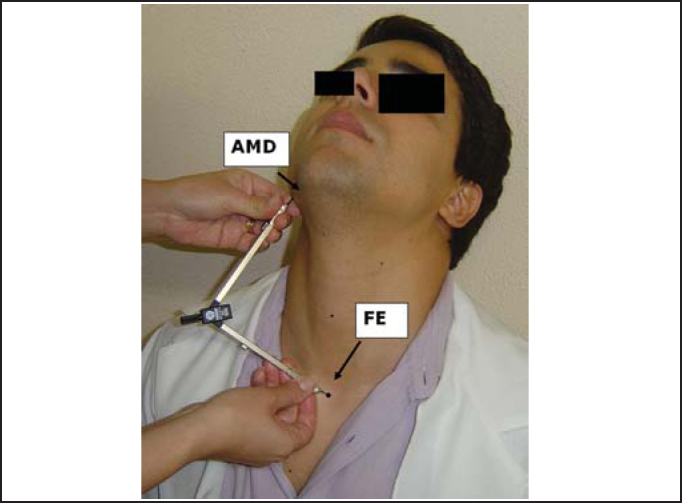
Marking of measurement of right mandible angle (AMD) to center of sternal furculum (FE).

The study gathered 68 young adults aged 18 to 44 years, 33 female and 35 male subjects. They were all healthy and did not present any vocal complaints or history of dysphonia.

Initially, participants recorded their voices to register fundamental frequency. The software used was GRAM, version 5.7. Subjects were instructed to produce a sustained vowel “e” standing up, with unidirectional microphone placed 10cm from the mouth of the speaker, at a 45º angle.

Measurements of laryngeal height were performed using a compass (a small rubber was adapted to the sharp tip to avoid accidents) and a 20cm ruler, when patients were sitting down and had their heads at maximum hyperextension.

The landmarks used for the measurements were angle of right mandible (AMD) and left mandible (AME), center of the arch of cricoid cartilage (CC) and sternal furculum center (FE). These points were properly marked by using a waterbase pen.

To reach the vertical position of the larynx in the neck, we measured the following distances: AMD-AME - from the right mandible angle to the left mandible angle; AMD-CC - from the right mandible angle to the center of the cricoid cartilage arch, and AMD-FE - from the right mandible angle to the center of the sternal furculum. Initially, measurements were made with the compass placed on the subject and then marked with the ruler.

Based on the measurements made, we had the values of length B/2, L and l. Heights H and h were obtained by applying Pythagoras Theorem in two rectangular triangles: the smaller one formed by AMD-Y, Y-CC and AMD-CC; and the larger one formed by AMD-Y, Y-FE and AMD-FE. Based on the result of the difference between H and h, we obtained the value of X that corresponded to the distance from CC to FE.

We defined that PVL would be a value relative to H, so:

Given X = H - h
H: Height of larger triangle

**Figure 5:**
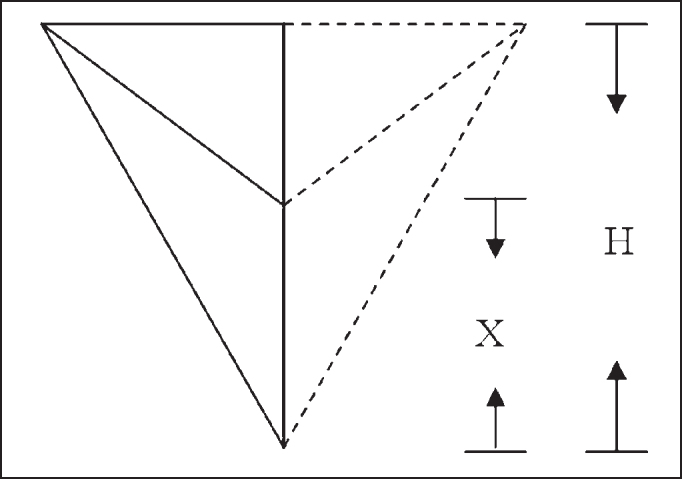
High PVL in the neck.

**Figure 6:**
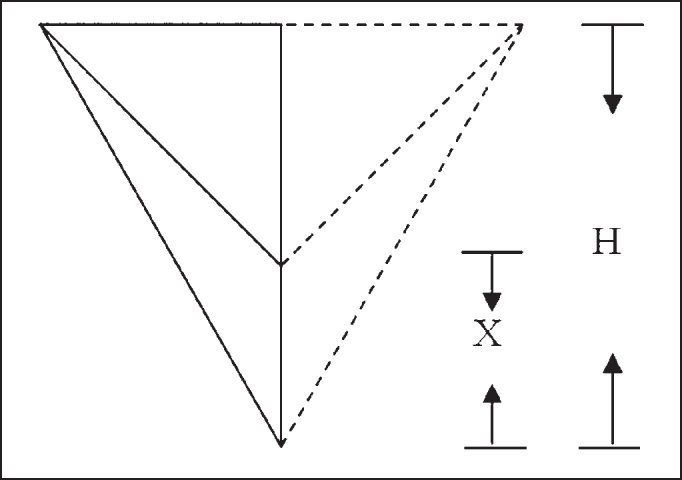
Low PVL in the neck.

Thus, the higher the PVL value, the more elevated was the larynx in the neck (Figure 5); the lower the PVL value, the lower the larynx was placed in the neck (Figure 6).

Statistical analysis was performed with *t Student test*. The adopted level of significance was 5%.

## RESULTS

Results are presented in the tables below.

## DISCUSSION

**Figure 4:**
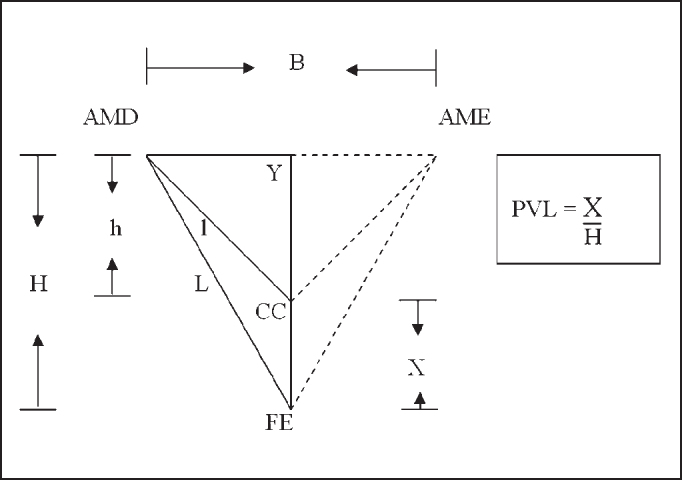
AMD-AME - Angle of right mandible up to left mandible angle AMD - CC - Right mandible angle up to center cricoid cartilage arch AMD - FE - Angle of the right mandible up to center of sternal furculum B - Height of AMD-AME l - Height of AMD - CC L - Height of AMD - FE h - Height of smaller triangle H - Height of larger triangle (H = h + X) X - Distance between two points CC and FE (X = H - h) Y - Average point AMD-AME.

Laryngeal vertical position (PVL) has significant acoustic and physiological implications. Variation of vocal frequency is a direct acoustic consequence of vertical movement of the larynx in the neck.

The elevated laryngeal position is a frequently found characteristic in hyperfunctional dysphonias1., 6.. The tension of laryngeal muscle may produce a series of vocal affections, such as aphonia, breathiness, hoarseness, excessively high pitch^1^. According to Angsuwarangsee and Morrison, dysphonia by muscle tension is probably the most common cause of non-organic origin of vocal affections^7^. Musculoskeletal tension reduction, reached by means of vocal techniques such as yawning and sighing, laryngeal manipulation and prolonged/b/, leads to vocal production of better quality. Lower positioning of a normally elevated larynx is sometimes a specific objective to be reached by singing classes and vocal clinical practice.

Measurement of laryngeal position at rest is technically difficult, especially because there are no fixed points that serve as a reference to obtain the measurements. Out of the few studies available to investigate the laryngeal position in the neck, most aimed at analyzing vertical laryngeal movement in speech, singing and breathing6., 8., 9.. Previous measurements of laryngeal position were obtained thanks to the use of optical devices, x-ray and photographic equipment8., 10.. Honda, Hirai, Masaki & Shimada (1999) used magnetic resonance imaging to analyze the role of laryngeal vertical movement and cervical lordosis in the control of vocal frequency^9^.

To standardize and prevent variations of head positions, we decided to make the measurements of participants with the head at maximum hyperextension. We decided to use this element to allow intra-subject comparisons during clinical follow-up. Some participants were excluded from the study because they had very bulky neck, hindering the identification of landmarks.

In our study, we defined PVL as the relative V at H (height of the larger triangle), because the statistical analysis showed that H had an important role in the differentiation between male and female gender. We also observed that PVL was highly correlated with the relation between l (height of AMD-CC) and L (length of AMD-FE), or in other words, the higher the value of PVL, the smaller the relation l/L. However, data showed that the relation l/L was not good to differentiate the genders.

Behavioral analysis of data did not reveal direct correlation between fundamental frequency measurement and PVL both in female gender (Table 1) and male gender (Table 2), probably because the study had been performed in normal subjects. Maybe this correlation can be observed in other studies that will compare normal and dysphonic subjects.

**Table 1:** Values of fundamental frequency measures and vertical position of young adult larynges in female subjects without vocal complaints

Subjects	Age (Years)	F_0_ (Hz)	PVL
1	27	210	0.46
2	21	199	0.47
3	22	210	0.39
4	22	210	0.44
5	20	210	0.49
6	36	220	0.51
7	25	188	0.66
8	32	177	0.53
9	24	188	0.46
10	31	220	0.50
11	22	231	0.51
12	27	220	0.42
13	37	177	0.41
14	42	199	0.46
15	24	210	0.37
16	22	220	0.48
17	23	220	0.41
18	28	199	0.40
19	22	210	0.51
20	27	199	0.54
21	19	220	0.46
22	25	188	0.44
23	21	242	0.48
24	18	188	0.42
25	33	210	0.44
26	24	199	0.44
27	23	220	0.52
28	36	177	0.58
29	19	210	0.45
30	23	188	0.41
31	23	231	0.42
32	26	242	0.42
33	40	220	0.32

**Table 2:** Values of fundamental frequency measures and vertical position of young adult larynges in male subjects without vocal complaints.

Subjects	Age (Years)	F_0_ (Hz)	PVL
1	23	102	0.41
2	29	123	0.50
3	27	102	0.39
4	29	156	0.43
5	19	134	0.46
6	32	113	0.41
7	36	123	0.45
8	36	123	0.40
9	23	113	0.50
10	30	134	0.44
11	19	134	0.39
12	26	91	0.41
13	32	145	0.37
14	40	91	0.39
15	44	113	0.45
16	29	134	0.45
17	22	91	0.43
18	28	102	0.40
19	27	113	0.46
20	19	134	0.40
21	26	113	0.43
22	28	113	0.35
23	24	113	0.44
24	29	145	0.37
25	30	156	0.44
26	29	123	0.48
27	24	134	0.53
28	33	123	0.39
29	22	134	0.44
30	31	113	0.37
31	20	102	0.46
32	27	113	0.52
33	30	123	0.37
34	28	102	0.43
35	26	113	0.38

Data obtained in this study confirmed the findings of the literature for normal subjects^3^. The position of the larynx in female patients proved to be statistically higher in male patients. Fundamental frequency measurement in female patients was significantly higher than in male subjects (Table 3), with values close to those reported by Behlau, Tosi and Pontes.^11^

**Table 3:** Mean of values of laryngeal vertical position and fundamental frequency measurement in young adults without vocal complaints.

	Female	Male	Value of p
Laryngeal Vertical Position	0.46	0.43	0.015*
Fundamental Frequency	207.64	119.74	<0.001*

^*^ Statistically significant difference

The present study allowed us to confirm that this form of measurement of the laryngeal vertical position can be performed and that it is an interesting parameter for intra-subject clinical follow-up of dysphonic patients, as well as for cases of incomplete vocal changes.

## CONCLUSION

The technique to reach vertical laryngeal position in both genders proved to be easy and practical. Female larynges were positioned higher than male larynges.
